# Application of the Strain Compensation Model and Processing Maps for Description of Hot Deformation Behavior of Metastable β Titanium Alloy

**DOI:** 10.3390/ma14082021

**Published:** 2021-04-17

**Authors:** Oleksandr Lypchanskyi, Tomasz Śleboda, Aneta Łukaszek-Sołek, Krystian Zyguła, Marek Wojtaszek

**Affiliations:** Faculty of Metals Engineering and Industrial Computer Science, AGH University of Science and Technology, Av. Mickiewicza 30, 30-059 Krakow, Poland; sleboda@agh.edu.pl (T.Ś.); alukasze@metal.agh.edu.pl (A.Ł.-S.); kzygula@agh.edu.pl (K.Z.); mwojtasz@metal.agh.edu.pl (M.W.)

**Keywords:** β titanium alloy, constitutive model, flow behavior, processing maps

## Abstract

The flow behavior of metastable β titanium alloy was investigated basing on isothermal hot compression tests performed on Gleeble 3800 thermomechanical simulator at near and above β transus temperatures. The flow stress curves were obtained for deformation temperature range of 800–1100 °C and strain rate range of 0.01–100 s^−1^. The strain compensated constitutive model was developed using the Arrhenius-type equation. The high correlation coefficient (R) as well as low average absolute relative error (AARE) between the experimental and the calculated data confirmed a high accuracy of the developed model. The dynamic material modeling in combination with the Prasad stability criterion made it possible to generate processing maps for the investigated processing temperature, strain and strain rate ranges. The high material flow stability under investigated deformation conditions was revealed. The microstructural analysis provided additional information regarding the flow behavior and predominant deformation mechanism. It was found that dynamic recovery (DRV) was the main mechanism operating during the deformation of the investigated β titanium alloy.

## 1. Introduction

In recent years, the use of metastable β titanium alloys has grown steadily due to their ability to process at lower temperatures, good corrosion resistance, higher fatigue strengths, as well as the possibility of increasing the level of yield strength by hardening. Because of such a set of properties, this type of alloy is widely used in the aerospace industry [1]. Among the β alloys, the high strength Ti-10V-2Fe-3Al (Ti-1023) alloy should be noted due to its high combination of mechanical properties and good workability, which has influenced its widespread use in aircraft landing gear components [1,2].

Two of the most expedient methods of hot plastic deformation for ensuring the necessary mechanical properties of critical parts made of titanium alloys are hot die forging and isothermal forging. In addition, in recent years, methods based on powder metallurgy have become widespread for obtaining finished products from titanium alloys [3,4]. The main parameters of plastic deformation, determining the changes in the microstructure and deformation behavior of titanium alloys during processing, are temperature, strain, and strain rate. In addition, significant influence of the initial microstructure on the flow behavior during hot forming of the Ti-1023 alloy was noted [2,5,6]. The initial morphology of the globular α phase has a less effect on the increase in flow stress during isothermal forging below β transus temperature, as compared to α lamellae with a high aspect ratio. The increased sensitivity of the microstructure of the Ti-1023 alloy in near β transus (about 800 °C) temperature [2] should also be noted.

Thermomechanical processing parameters have a significant impact on the changes occurring in the microstructure during deformation of the materials that show susceptibility to dynamic recrystallization (DRX) or dynamic recovery (DRV). The identification of these changes in the microstructure has been the aim of many studies in the field of constitutive and dynamic material modeling for describing the hot deformation behavior of both metallic materials and in particular β titanium alloys [7,8,9,10,11,12,13,14,15]. Xiao et al. [7] investigated the hot deformation flow behavior of a Ti-55511 alloy during hot compression using dislocation density-based constitutive model as well as processing maps. Moreover, Lin et al. [11] showed a comparative analysis of artificial neural network, Hensel-Spittel, and strain-compensated Arrhenius-type models to describe the flow behavior of Ti-55511 titanium alloy. Fan et al. [9] and Wu et al. [12] described the deformation mechanisms of Ti-7Mo-3Nb-3Cr-3Al and Ti-4Al-1Sn-2Zr-5Mo-8V-2.5Cr alloys, respectively, based on processing maps and constitutive equations. Zhao et al. [13] and OuYang et al. [14] analyzed the deformation mechanisms of Ti-1023 alloy during hot compression at supertransus temperatures using activation energy and DRX kinetics model, respectively. The hot flow behavior of Ti-1023 alloy has also been analyzed by the artificial neural network model, the constitutive model using regression method, and the physically-based constitutive model [15,16]. Besides, one of the hot deformation optimization approaches for Ti-1023 alloy was the use of processing maps based on dynamic materials modeling (DMM) [17,18]. Superimposing hot processing maps over flow stress maps as well as overactivation of energy values to evaluate the hot workability of the investigated materials is quite often applied [19,20].

The models based on Arrhenius-type constitutive equation are the most widely used phenomenological constitutive models that have been successfully used for descriptions of the flow behavior during hot deformation of alloys and metals [8,11,21,22,23,24,25]. The constitutive equation describing the flow behavior of Ti-1023 alloy obtained by the blended elemental powder metallurgy technique was elaborated and presented in [26]. The highest accuracy of the Arrhenius-type equation in the form of a hyperbolic sine law was confirmed in this respect. However, it should be noted that this equation does not take into account the influence of the strain level on the flow behavior of the material, which is an important factor for designing the technological parameters of hot working. It is worth emphasizing that most of the research works were focused on the hot deformation behavior of Ti-1023 below or near β transus temperature ranges. On the other hand, the flow behavior of this alloy in β region is not fully understood, which makes this issue worthy of deeper analysis. It is known that deformation above β transus temperature leads to DRX and prior refinement of β grains, and therefore finer lamella microstructure increasing the ductility as well as strength of this alloy [14,27].

For a deeper understanding of the high-temperature workability of Ti-1023 alloy, the results of flow behavior at near and above β transus temperature range are presented in this paper. Based on hot compression tests, the strain compensated Arrhenius-type model and processing maps were developed and discussed with regard to changes in the microstructure of the Ti-1023 alloy.

## 2. Materials and Methods

The chemical composition of the investigated titanium alloy was Ti-9.76V-1.84Fe-3.37Al (wt.%). The size of cylindrical specimens for hot compression tests was 10 mm in diameter and 12 mm in height. The specimens were machined from cast alloy rod (ϕ76 mm) and deformed in compression on the Gleeble 3800 thermomechanical simulator (Dynamic Systems, Inc., Poestenkill, NY, USA) to a total true strain of 1. The specimens were cut along the rod axis at a distance of approx. 2/3 of the radius from the center of the rod cross-section. The isothermal tests were performed under an argon atmosphere at the temperature varying from 800 °C to 1100 °C, and at strain rates of 0.01 s^−1^, 0.1 s^−1^, 1 s^−1^, 10 s^−1^, and 100 s^−1^. A graphite foil was used as a lubricant in order to minimize the friction effect between the specimen and anvils. The samples were homogenized in 10 s prior to deformation and the resistance heating rate of 2.5 °C/s was applied. After compression, the samples were cooled in air and machined along the axial direction for microstructure observation.

**Figure 1:**
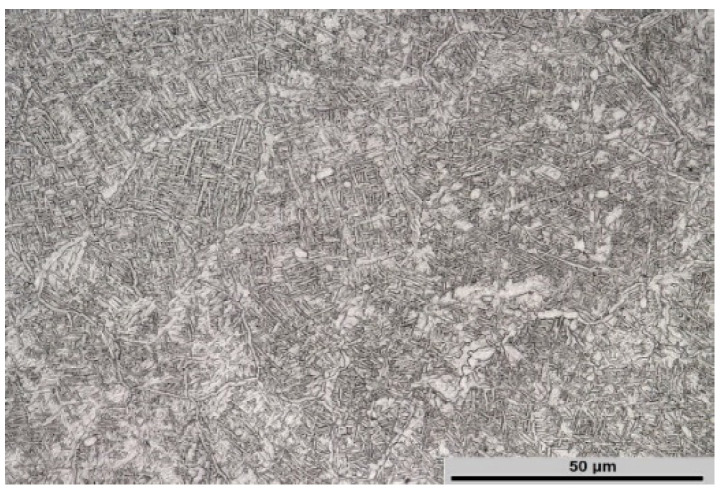
Starting microstructure of Ti-1023 alloy.

The microstructure of Ti-1023 alloy in the as-received condition (Figure 1) consists of original β grains, intragranular lamellar α colonies with high volume fraction and continuous α layers on the β grains boundaries.

## 3. Results and Discussion

### 3.1. Flow Behaviour

It is well known that the influence of friction as well as adiabatic heating on flow stresses during compression tests leads to deviations in flow stress and difficulties associated with their interpretation. Taking into account this fact, the correction for friction and heating during deformation in order to precisely describe flow behavior of the material is an important factor. In the presented studies, the inverse analysis technique [28,29] was used in order to correct the data obtained on the basis of compression tests.

**Figure 2:**
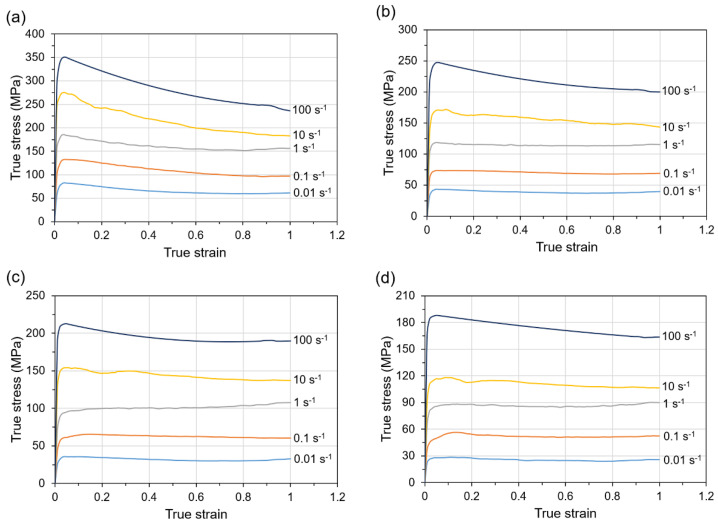

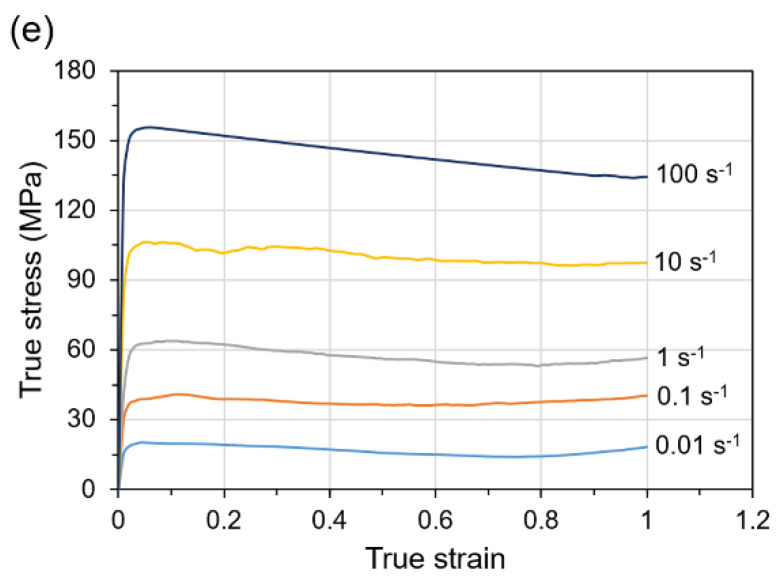
The true stress-true strain curves for Ti-1023 alloy deformed in compression at the temperature of (**a**) 800 °C, (**b**) 900 °C, (**c**) 950 °C, (**d**) 1000 °C, (**e**) 1100 °C and at various strain rates.

Based on the isothermal hot compression tests, true stress-strain curves for Ti-1023 alloy deformed to a true strain of 1 at the temperature range of 800–100 °C, and strain rate range of 0.01–100 s^−1^ (Figure 2) were obtained.

Generally, the expected decrease of flow stress with increasing temperature and decreasing strain rate was observed. As can be seen from the obtained curves, they are characterized in most of the cases by steady state flow after reaching a peak value at the initial stage. This type of material flow behavior can mostly be noticed in the case of materials subjected to deformation at the temperatures higher than supertransus temperature and is quite typical for β titanium alloys processed at this temperature range [30,31]. Rapid work hardening and subsequent flow softening effects during deformation at the temperature below β transus temperature (Figure 2a) indicated the occurrence of DRX or DRV. However, in this case, it should be noted that the flow curves obtained for the material deformed at strain rate of 10 s^−1^ and 100 s^−1^ show flow softening followed by continuous decrease of the flow stress without steady state flow region, and саn indicate flow instability under such deformation conditions. It should also be noted that most of the curves, especially obtained at low strain rates (≥1 s^−1^), do not have distinct dynamic softening effects, which indicate the occurrence of the predominant DRV mechanism during deformation.

### 3.2. Development of the Constitutive Equation

The high-temperature deformation behavior of the material can be described by the relationships of the strain rate, temperature and flow stress, e.g., by the Arrhenius-type equation [25,32]:

(1) $$\dot{\varepsilon }=AF\left(\sigma \right)\exp\left(-\frac{Q}{RT}\right)$$

where *σ* is the flow stress (MPa), $\dot{\varepsilon }$ is the strain rate (s^−1^), R is the universal gas constant (8.314 J∙mol^−1^∙K^−1^), *A* is the material constant, *Q* is the deformation activation energy (kJ∙mol^−1^), *T* is the deformation temperature (K), and *F*(*σ*) is the flow stress function.

Depending on the stress levels, the flow stress function in Equation (1) can be expressed as given in [21]:

(2) $$\dot{\varepsilon }=A_{1}\sigma^{n_{1}}\exp\left(-\frac{Q}{RT}\right),\;\mathrm{for}\;\alpha \sigma <0.8$$

(3) $$\dot{\varepsilon }=A_{2}\exp\left(\beta \sigma \right)\exp\left(-\frac{Q}{RT}\right),\;\mathrm{for}\;\alpha \sigma >1.2$$

(4) $$\dot{\varepsilon }=A{\left[\sin h\left(\alpha \sigma \right)\right]}^{n}\exp\left(-\frac{Q}{RT}\right),\mathrm{for}\;\mathrm{all}\;\sigma$$

where *A*, *A*_1_, *A*_2_, *n*_1_, *β*, *n* and *α* are the material constants, *α = β/n*_1_.

The relationship between the strain rate and temperature is described by the Zener-Hollomon parameter (*Z*) [33]:

(5) $$Z=\dot{\varepsilon }\exp\left(\frac{Q}{RT}\right)$$

After transformation of Equations (2)–(4) into the natural logarithm, they allow to calculate the materials’ constants:

(6) $$\ln\dot{\varepsilon }=\ln A_{1}-\frac{Q}{RT}+n_{1}\ln\sigma$$

(7) $$\ln\dot{\varepsilon }=\ln A_{2}-\frac{Q}{RT}+\beta \sigma$$

(8) $$\ln\dot{\varepsilon }=\ln A-\frac{Q}{RT}+n\ln\left[\sin h\left(\alpha \sigma \right)\right]$$

**Figure 3:**
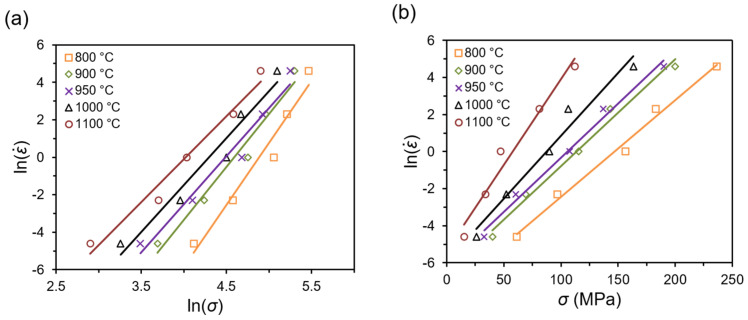

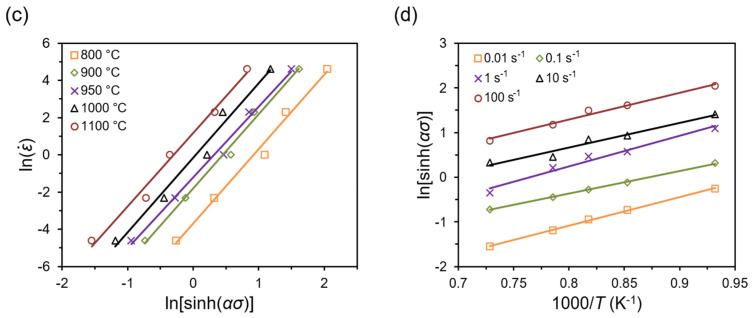
Relationships between (**a**) ln$\dot{\varepsilon }$ and ln*σ*, (**b**) ln$\dot{\varepsilon }$ and *σ*, (**c**) ln$\dot{\varepsilon }$ and ln[sinh(*ασ*)], (**d**) ln[sinh(*ασ*)] and (1/*T*) for a true strain of 1.

According to Equations (6)–(8), using the corresponding strain rates and peak flow stresses data, the relationships of ln$\dot{\varepsilon }$-ln*σ* (Figure 3a), ln$\dot{\varepsilon }$-*σ* (Figure 3b) as well as ln$\dot{\varepsilon }$-ln[sinh(*ασ*)] (Figure 3c) can be plotted. The average linear slopes on the obtained diagrams allow determining the material constants *n*_1_, *β*, and *n* as 5.405, 0.0626, and 3.942, respectively, as well as *α* parameter (0.0116). Based on the transformation of Equation (8) and the subsequent average slopes of linear regression lines of ln[sinh(*ασ*)]-(1/*T*) relationships presented in Figure 3d, the deformation activation energy *Q* can be determined as 195.682 kJ/mol. The presented activation energy value for Ti-1023 alloy deformed near and above β transus temperatures is quite close to the activation energy presented for that type of titanium alloy deformed in compression at β phase temperature ranges noted in other studies 172 kJ/mol [13] and 210.45 kJ/mol [15] for Ti-1023 alloy or 222.173 kJ/mol for Ti-555211 alloy [34]. It should be noted that the calculated activation energy for the β phase region is significantly lower as compared to the α + β phase region for β titanium alloys. In addition, the obtained calculated Q value for the investigated alloy is slightly higher as compared to the self-diffusion activation energy for β titanium (153 kJ/mol) or α titanium (169 kJ/mol) [35,36]. It is generally accepted that the calculated Q value is much higher than the value of self-diffusion energy, indicating the presence of globularization or DRX as a predominant mechanism during deformation, while closer activation energy value is a characteristic feature for DRV [37]. In this regard, it should be considered that DRV is the predominant deformation mechanism for Ti-1023 alloy at temperatures above β transus temperature. This fact is also confirmed by the analysis of the flow stress curves (Figure 2), which in most of the cases showed steady state flow of the material without distinct dynamic softening effects.

After the conversion to logarithmic form, Equation (5) can be written as:

(9) lnZ=lnA+nln[sinh(ασ)]

**Figure 4:**
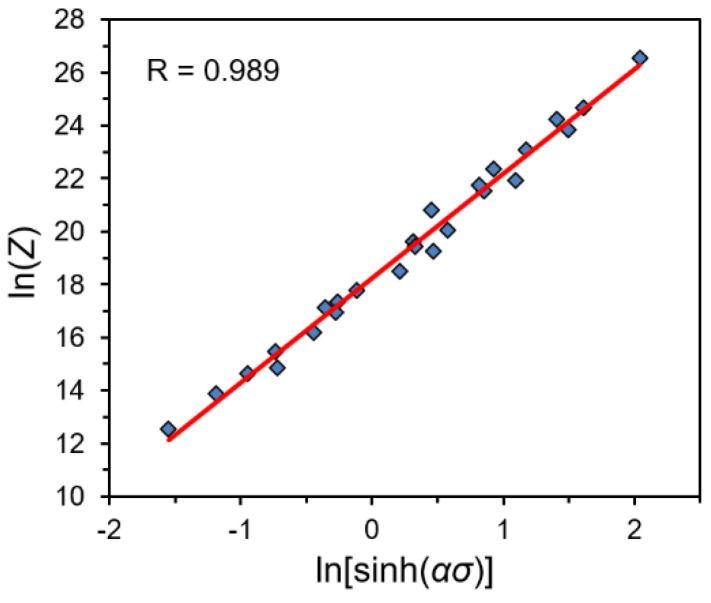
The relationship between ln*Z* and ln[sinh(*ασ*)].

From the relationship between ln*Z* and ln[sinh(*ασ*)] (Figure 4) obtained using Equations (5) and (9), the ln*A* (18.243) was calculated by linear regression with high correlation coefficient (*R* = 0.989). In addition, the obtained fitting curve also allows to determine the *n* value as 3.934, and the small deviations between both presented values confirms the accuracy of both calculation methods.

Basing on the hyperbolic law, the flow stress as a function of the Zener-Hollomon parameter variable can be expressed as:

(10) σ=1αln{(ZA)1n+[(ZA)2n+1]12}

Taking into account the previously calculated material constants *n*, *α*, and *A*, the equation describing the flow stress at a constant true strain value can be given as:

(11) σ=10.0116ln{(Z8.372×107)13.942+[(Z8.372×107)23.942+1]12}

### 3.3. Strain Compensated Constitutive Model

**Figure 5:**
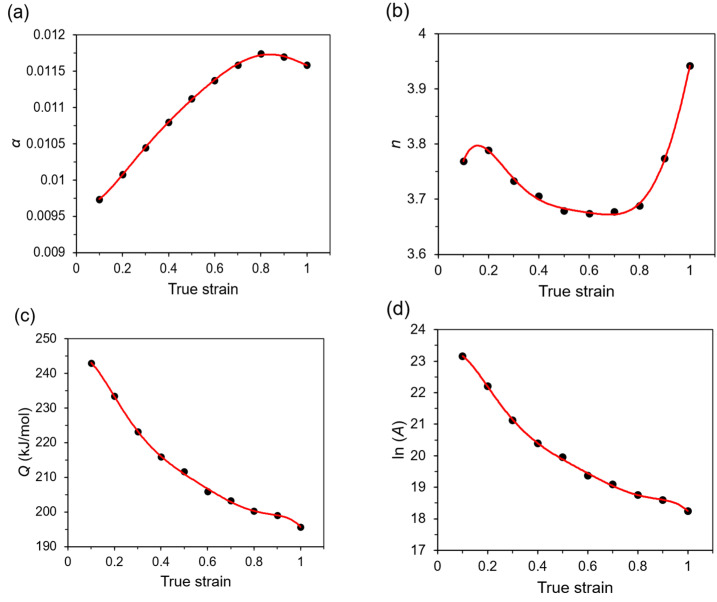
Material parameters (**a**) α, (**b**) *n*, (**c**) *Q*, and (**d**) ln*A* for various true strain levels.

One of the main deformation parameters that affect the DRV and DRX mechanisms is a true strain. During hot compression, the true stress-true strain relationship describes the flow behavior of the material, inter alia work hardening, and flow softening effects. It is also known that the material parameters also depend on strain level, which was not taken into account before this stage of this research. The compensation of strain for description of hot deformation behavior can be considered basing on the polynomial functions of the strain for the given material parameters [25,38]. In these studies, the polynomial function was used to describe the influence of *α*, *n*, *Q*, and *A* material parameters on true strain range of 0.1 to 1, at 0.1 interval (Figure 5), for the investigated temperatures and strain rates. It was determined that the 6th-order polynomial function (Equation (12)) is the most optimal for describing variable material parameters as a function of true strain.

(12) $$\left\{ \begin{array}{c} \alpha =B_{0}+B_{1}\varepsilon +B_{2}\varepsilon^{2}+B_{3}\varepsilon^{3}+B_{4}\varepsilon^{4}+B_{5}\varepsilon^{5}+B_{6}\varepsilon^{6}\\ n=C_{0}+C_{1}\varepsilon +C_{2}\varepsilon^{2}+C_{3}\varepsilon^{3}+C_{4}\varepsilon^{4}+C_{5}\varepsilon^{5}+C_{6}\varepsilon^{6}\\ Q=D_{0}+D_{1}\varepsilon +D_{2}\varepsilon^{2}+D_{3}\varepsilon^{3}+D_{4}\varepsilon^{4}+D_{5}\varepsilon^{5}+D_{6}\varepsilon^{6}\\ lnA=F_{0}+F_{1}\varepsilon +F_{2}\varepsilon^{2}+F_{3}\varepsilon^{3}+F_{4}\varepsilon^{4}+F_{5}\varepsilon^{5}+F_{6}\varepsilon^{6} \end{array}\right.$$

The coefficients (*B_i_*, *C_i_*, *D_i_* and *F_i_*, for *i* = 0–6) for Equation (12) were obtained from the polynomial approximation (red lines) of the material parameters presented in Figure 5, and are given in Table 1.

**Figure 6:**
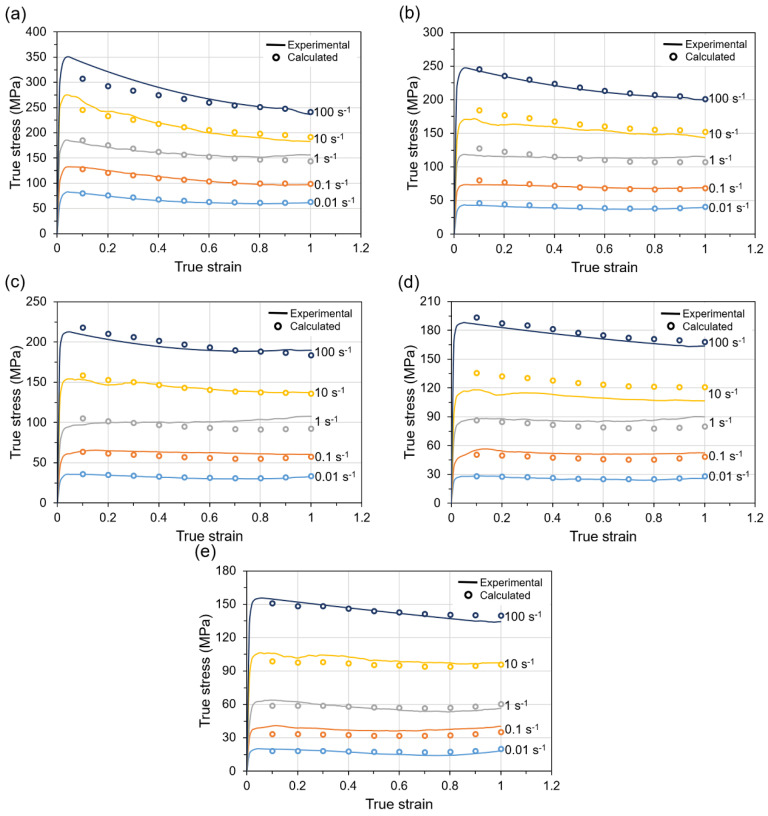
Experimental and calculated flow stresses for Ti-1023 alloy for deformation temperatures of (**a**) 800 °C, (**b**) 900 °C, (**c**) 950 °C, (**d**) 1000 °C, (**e**) 1100 °C.

After determining the material parameters as a function of true strain, flow stresses were calculated taking into account the strain compensated constitutive model adopted for description of hot deformation behavior of Ti-1023 alloy at the investigated temperatures, strain and strain rate ranges. Figure 6 presents the experimental flow stresses obtained during hot compression tests and the flow stresses calculated on the basis of the constitutive equations and also taking into account various strain levels. As can be seen in Figure 6, there is a high correlation between the calculated and experimental flow stress values.

The verification of the developed strain compensated model is based on discrepancies between experimental and calculated flow stress data. The most indicative parameters of data prediction are the correlation coefficient (*R*) and an average absolute relative error (*AARE*, %), that can be determined by Equations (13) and (14), respectively [11].

(13) $$R=\frac{\sum_{i=1}^{N}\left(E_{i}-\bar{E}\right)\left(P_{i}-\bar{P}\right)}{\sqrt{\sum_{i}^{N}{\left(E_{i}-\bar{E}\right)}^{2}{\left(P_{i}-\bar{P}\right)}^{2}}}$$

(14) $$AARE=\frac{1}{N}\overset{N}{\underset{i=1}{\sum }}\left|\frac{E_{i-}P_{i}}{E_{i}}\right|\times 100\%$$

where *E_i_* is experimental flow stress and *P_i_* is calculated flow stress, $\bar{E}$ and $\bar{P}$ are the mean values betwen experimental and calculated flow stress, *N* is the total number of data.

**Figure 7:**
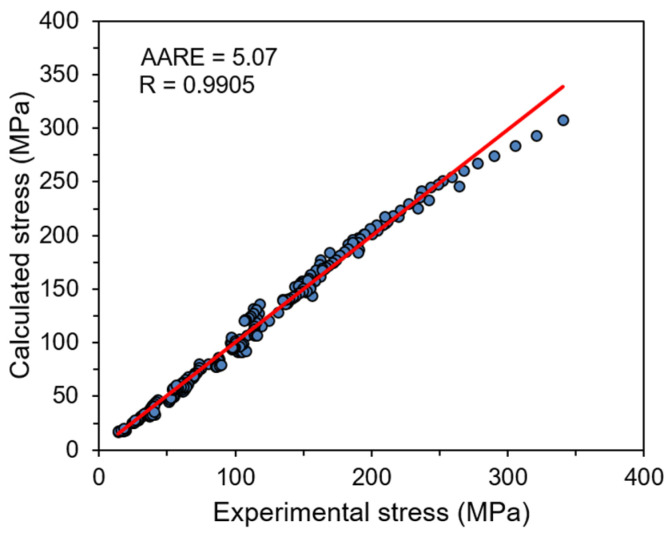
The correlation between calculated and experimental flow stresses for Ti-1023 alloy.

It is generally accepted that when the value of *R* coefficient is closer to 1 with small *AARE* value, then higher accuracy of flow stress calculations can be obtained. The developed strain compensated constitutive model describing flow behavior of Ti-1023 alloy is characterized by *R* value of 0.9905 and the low *AARE* (5.07%) level. As can be seen in Figure 7, the relationship between calculated and experimental data points is almost linear, which confirms the high accuracy of the developed model.

### 3.4. Processing Maps

The approaches based on DMM theory using criteria determining the material flow instability are an effective tool for describing the hot workability of the materials. The stability criterion developed by Prasad has shown its effectiveness in predicting the deformation behavior of many alloys [4,39,40,41,42,43,44]. The distribution of power dissipation and flow instability parameters in the form of maps allows controlling changes in microstructure during the deformation of the investigated material. The fundamental parameter in DMM is the efficiency of power dissipation (*η*), which indicates the power dissipated through microstructural development mechanisms such as phase transformations, DRV or DRX [40,42,45] and can be determined as:

(15) $$\eta =\frac{2m}{m+1}$$

where *m* is the strain rate sensitivity parameter, which can be defined as a function of strain rate $\dot{\varepsilon }$ ($m=\partial \;\log\sigma /\partial \;\log\dot{\varepsilon })$.

It is well known that a higher value of the *η* parameter corresponds to a better hot workability of the material due to a larger share of the power dissipated resulting from changes in the material microstructure. On the other hand, the flow instability during hot deformation of the material can be described using the criterion (*ξ*) proposed by Prasad [42,44]:

(16) $$\xi \left(\dot{\varepsilon }\right)=\frac{\partial \ln\left(\frac{m}{m+1}\right)}{\partial \ln\;\dot{\varepsilon }}+m\leq 0$$

Negative vales of instability criterion parameter *ξ* reflects the instability of the material flow that can be characterized by adiabatic shear bands, kink bands, Lüders bands, flow localization, or cracking [43,46]. The superimposition of power dissipation map on instability map allows to develop the processing map for the investigated deformation parameters. Basing on the processing maps, it is possible to optimize the thermomechanical processing parameters and the material flow behavior can be predicted.

**Figure 8:**
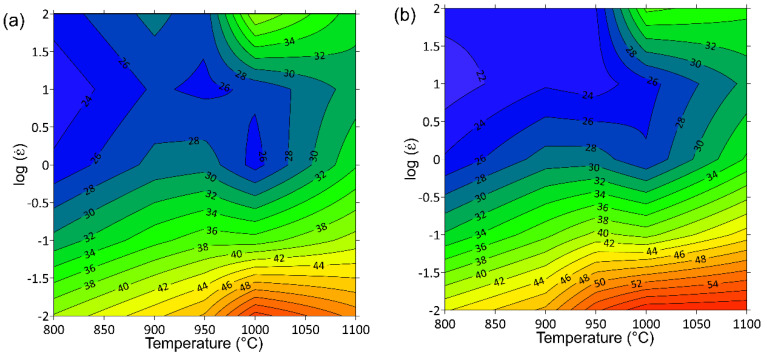

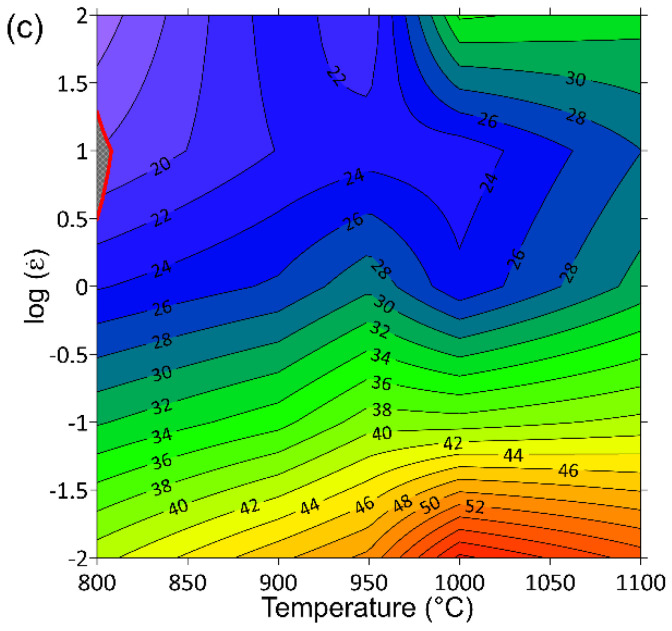
Processing maps for Ti-1023 alloy developed for true strains of (**a**) 0.2, (**b**) 0.6, and (**c**) 1.

Basing on the DMM as well as on Prasad stability criterion, the processing maps for the deformation temperature range from 800 °C to 1100 °C and strain rate range of 0.01–100 s^−1^, and for true strains 0.2, 0.6 and 1 (Figure 8) were developed. These maps represent the distribution of *η* parameter expressed as a percentage (black isoclines) and instability criterion *ξ* ≤ 0 (gray shaded areas with red borders). The nature of the distribution of the efficiency of power dissipation indicates that the hot workability of the investigated titanium alloy is better at lower strain rates, which is typical for many titanium alloys and particularly for β titanium alloys [7,9,47,48]. As can be seen in Figure 8, the tendency of the distribution of the efficiency of power dissipation does not change with the change of the true strain value. It is generally accepted that the value of the efficiency of power dissipation in the range of 20–35% indicates DRV, while more than 35% is associated with DRX or superplasticity [7]. The high values of the *η* parameter can also indicate high ductility of large grains caused by substructure formation mechanisms [49,50]. As can be seen from the distribution of power dissipation, the DRV is the predominant deformation mechanism at the investigated deformation conditions.

The distribution of criterion *ξ* shows that the flow instability (a negative values of *ξ*) appears only in a small area for the true strain of 1 (Figure 8c), at strain rates from 3 s^−1^ to 6 s^−1^ and deformation temperatures in the range of 800–810 °C. This means that the flow behavior of Ti-1023 is quite stable under the investigated deformation conditions. It should also be emphasized that the noted instability area is characterized by the low efficiency of power dissipation (21–17%), and it is also one of the reasons not to recommend the hot deformation under parameters corresponding to this range of processing parameters.

Based on the distribution of flow instability parameter *ξ* as well as the *η* parameter, the processing windows describing the most useful combinations of hot deformation parameters can be determined. First of all, a domain should be distinguished (first processing window), with peak values of the efficiency of power dissipation in the range from 56% to 60%, in the temperature range from 950 °C to 1050 °C and low (≥0.04 s^−1^) strain rates. The second processing window is located in the domain at the temperature range of 980–1050 °C and strain rates ≤ 60 s^−1^ with peak value of parameter *η* in the range of 38–34%. The hot workability of Ti-1023 alloy can be improved by the application of deformation conditions corresponding to the proposed process windows.

### 3.5. The Microstructure Evolution

**Figure 9:**
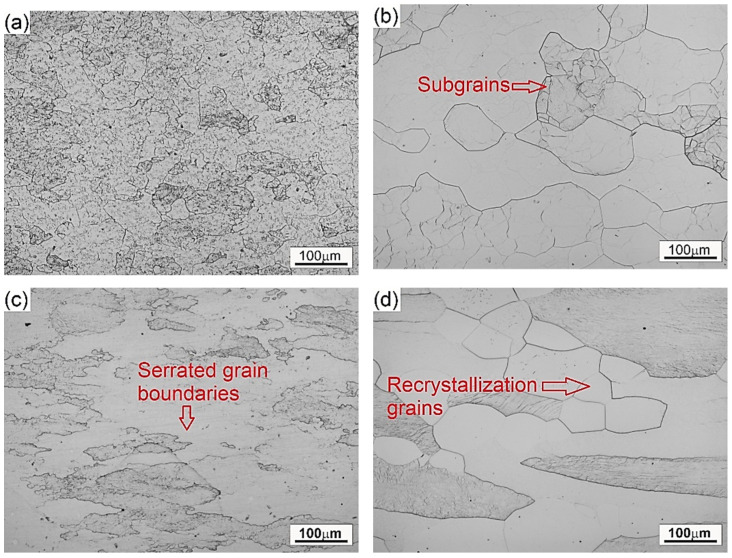
The microstructures of Ti-1023 alloy deformed under chosen processing conditions: (**a**) 800 °C and 0.01 s^−1^, (**b**) 1000 °C and 0.01 s^−1^, (**c**) 900 °C and 1 s^−1^, (**d**) 1100 °C and 100 s^−1^.

Figure 9 presents the microstructures of the specimens deformed in compression under various deformation temperatures and strain rates. The microstructure of the material deformed at temperature of 800 °C and strain rate of 0.01 s^−1^ (Figure 9a) is characterized by fine α phase precipitates, still remaining in the microstructure of the original β grains. An increase in the deformation temperature to 1000 °C (Figure 9b) leads to the complete dissolution of the α phase precipitates and to the effects associated with the DRV. The grain growth and deformation of the original β grains as well as the subsequent formation of the substructure as a result of deformation are observed. Such changes in the microstructure can be associated with superplasticity of large grains at low deformation strain rates, which was also reported in other studies [49,51]. In addition, the high value of parameter *η* (56–60%) indicates better workability during such deformation conditions as a result of a greater power dissipated due to the microstructural changes. The microstructure of the material deformed in the β phase region at temperature of 900 °C and strain rate of 1 s^−1^ is presented in Figure 9c. An increase in strain rate causes the original β grains to elongate in the direction of the material flow and the β grains boundaries are serrated. This effect is associated with DRV and affects the further nucleation of grains at the prior β phase boundaries, which is also visible in Figure 9c and is typical for DRX. At the highest deformation temperature and highest strain rate (Figure 9d), recrystallized grains and partial recrystallization of the microstructure can be observed.

**Figure 10:**
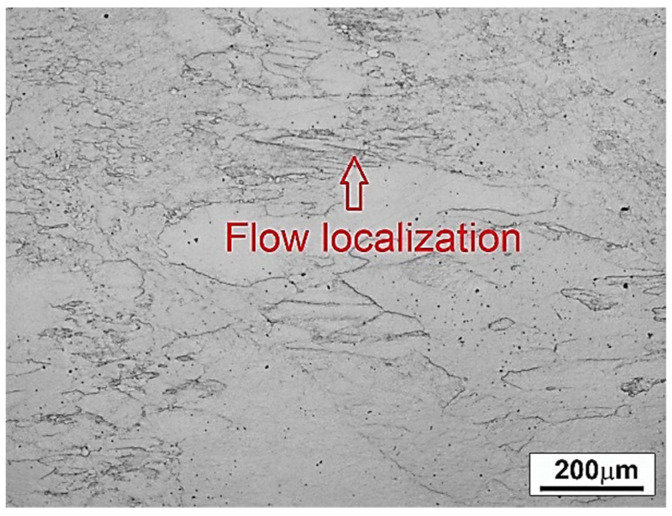
The microstructure of Ti-1023 alloy deformed under thermomechanical parameters corresponding to flow instability area.

As can be seen in Figure 10, the microstructure of the material deformed at 800 °C and under strain rate of 10 s^−1^ (conditions corresponding to instability area at processing map developed according to the Prasad criterion) indicates instability of the material flow (flow localization). It should be noted that the microstructure also contains, in addition to β grains, small particles of the α phase. It is known that inhomogeneous deformation of β grains can be associated with dislocations stuck at the interface between the α + β and β phases at high strain rates [47]. Taking into account the above analysis, the hot workability of Ti-1023 alloy under processing parameters corresponding to the presented flow instability is not recommended.

## 4. Conclusions

The analysis of deformation behavior of β Ti-1023 alloy at temperatures near and above β transus temperature leads to the following conclusions:

- Basing on the Arrhenius-type equation, the strain compensated constitutive model was developed for the description of the flow behavior of the investigated alloy during high-temperature deformation. Very low value of average absolute relative error and high correlation between calculated values of flow stress and experimentally obtained flow stresses confirmed a high accuracy of the developed model.
- The processing maps were generated upon the Prasad stability criterion for the investigated deformation conditions. The most favorable parameters of the alloy processing, as well as the areas of instability of the material flow, have been established. Generally, the high material flow stability was revealed.
- It was confirmed that the dynamic recovery is the main mechanism operating during the high-temperature deformation of Ti-1023 alloy. The analysis of the microstructure of the material deformed under the assumed thermomechanical conditions showed that DRV mechanisms, such as formation of subgrains or formation of serrated grain boundaries, play an important role in deformation behavior of this alloy.

## Figures and Tables

**Table 1:** The coefficients of material parameters *α*, *n*, *Q*, and *A* obtained basing on polynomial fitting.

*α*	*n*	*Q*, kJ/mol	ln*A*
*B*_0_ = 0.0097	*C*_0_ = 3.4595	*D*_0_ = 240.38	*F*_0_ = 22.69
*B*_1_ = −0.0027	*C*_1_ = 5.8104	*D*_1_ = 161.47	*F*_1_ = 20.393
*B*_2_ = 0.0411	*C*_2_ = −35.627	*D*_2_ = −1874.9	*F*_2_ = −215.18
*B*_3_ = −0.1249	*C*_3_ = 97.715	*D*_3_ = 6089.3	*F*_3_ = 687.83
*B_4_* = 0.1933	*C_4_* = −136.94	*D_4_* = −9574.8	*F_4_* = −1070.8
*B*_5_ = −0.1502	*C*_5_ = 94.922	*D*_5_ = 7379.5	*F*_5_ = 817.86
*B*_6_ = 0.0453	*C*_6_ = −25.4	*D*_6_ = −2225.2	*F*_6_ = −244.58

## Data Availability

Data sharing is not applicable to this article.
